# Developmental Competence of Domestic Cat Vitrified Oocytes in 3D Enriched Culture Conditions

**DOI:** 10.3390/ani9060329

**Published:** 2019-06-07

**Authors:** Martina Colombo, Maria Giorgia Morselli, Mariana Riboli Tavares, Maricy Apparicio, Gaia Cecilia Luvoni

**Affiliations:** 1 Dipartimento di Scienze Veterinarie per la Salute, la Produzione Animale e la Sicurezza Alimentare “Carlo Cantoni”, Università degli Studi di Milano, Via Celoria, 10, 20133 Milano, Italy; martina.colombo@unimi.it (M.C.); cecilia.luvoni@unimi.it (G.C.L.); 2 Departamento de Medicina Veterinária Preventiva e Reprodução Animal, Universidade Estadual Paulista (UNESP), Via de Acesso Prof. Paulo Donato Castellane s/n, Jaboticabal 14884-900, Brazil; nanariboli@yahoo.com.br (M.R.T.); maricyap@hotmail.com (M.A.)

**Keywords:** alginate microcapsule, blastocyst, cryopreservation, feline, in vitro culture, three-dimensional

## Abstract

**Simple Summary:**

Oocyte vitrification is a cryopreservation method that guarantees the long-term conservation of genetic material and fertility potential in humans and wild or domestic animals. However, in the domestic cat the in vitro embryo development of immature vitrified oocytes is not yet satisfactory. In this study, a three-dimensional (3D) culture system was used for the in vitro embryo production of vitrified oocytes to provide conditions more similar to those of the in vivo microenvironment, and for comparison, control vitrified oocytes were cultured in two-dimensional (2D) conditions. Embryos were cultured for seven days and their development was assessed. The results showed that the 3D enriched culture system was able to sustain the in vitro maturation and the subsequent embryo development of vitrified oocytes, but no differences were found with the 2D system and improvements to enhance the development of vitrified oocytes are still needed.

**Abstract:**

Cryoinjuries severely affect the competence of vitrified oocytes (VOs) to develop into embryos after warming. The use of culture conditions that provide physical and chemical support and resemble the in vivo microenvironment in which oocytes develop, such as 3D scaffolds and coculture systems, might be useful to improve VOs outcomes. In this study, an enriched culture system of 3D barium alginate microcapsules was employed for the in vitro embryo production of domestic cat VOs. Cryotop vitrified-warmed oocytes were in vitro matured for 24 h in the 3D system with or without fresh cumulus-oocyte complexes (COCs) in coculture, whereas a control group of VOs was cultured in traditional 2D microdrops of medium. After in vitro fertilization, presumptive embryos were cultured in 3D or 2D systems according to the maturation conditions. Vitrified oocytes were able to mature and develop into embryos in 3D microcapsules (17.42 ± 11.83%) as well as in 2D microdrops (14.96 ± 8.80%), but the coculture with companion COCs in 3D resulted in similar proportions of VOs embryo development (18.39 ± 16.67%; *p* = 1.00), although COCs presence allowed for blastocyst formation (0.95 ± 2.52%). In conclusion, embryos until late developmental stages were obtained from cat VOs, and 3D microcapsules were comparable to 2D microdrops, but improvements in post-warming conditions are still needed.

## 1. Introduction

Vitrification has widely improved oocyte cryopreservation techniques [1], but successful meiosis resumption and further embryo development of immature vitrified oocytes (VOs) are still difficult to achieve compared to fresh gametes [2]. Sub-zero temperatures, high concentrations of toxic cryoprotectants, and osmolality differences between the extra- and the intra-cellular environments [3] result in severe cryoinjuries on both germinal and somatic compartments [2]. Cumulus-cells loss limits VOs possibilities to fully mature after warming [4], cytoskeleton damages lead to the disassembly of the meiotic spindle and DNA alterations [5,6,7], and zona pellucida hardening may hinder sperm penetration [8]. As a consequence, the developmental competence of VOs after the restoration of physiological conditions is severely impaired [2]. Therefore, to preserve in toto the fertility potential and the reproductive performances, more specific and enriched culture conditions for stressed gametes, such as the VOs, are highly necessary.

In vivo, oocytes grow in a complex environment where cell-to-cell contacts, communications, and stimulating signaling allow female gametes to acquire their developmental competence. In vitro, to provide a more physiological environment for oocytes and embryos, three-dimensional (3D) culture systems have been applied with success. These systems, named scaffolds, were created and used for in vitro culture to provide a more suitable environment for cell growth, differentiation, and function [9], as they can trap cells or allow their migration and incorporate biologically active molecules [10,11,12]. Alginate scaffolds were successfully employed for oocyte, follicle, or embryo 3D culture and better meiosis resumption and embryonic developmental rates, as well as a genetic expression more similar to that observed in vivo, were reported [13,14,15,16,17].

To ameliorate oocytes performances in vitro, other successful attempts have been made using coculture systems, so that the presence of somatic cell monolayers or competent cumulus-oocyte complexes (COCs) gave physical (structural support) or chemical (secreted paracrine factors) improvements, which are beneficial for cocultured oocytes [18,19,20,21]. The association of 3D systems and cocultures was applied with good results for fresh domestic cat oocytes [22,23], but for VOs, satisfactory improvements in terms of maturation and cleavage rates have only been documented in 2D coculture in other species (pig [24,25], buffalo [26]).

In this study, enriched culture conditions were applied for the in vitro embryo production of low-competence cat VOs to investigate whether they might be suitable for their development. A 3D environment of barium alginate microcapsules (physical factor) alone or in association with fresh homologous COCs (chemical factors) was used during in vitro maturation of VOs, followed by in vitro fertilization and in vitro embryo culture in a 3D system.

## 2. Materials and Methods

### 2.1. Chemicals and Reagents

All chemicals and reagents were purchased from Sigma Chemical Company (St. Louis, MO, USA), unless otherwise stated.

### 2.2. Animals and Oocyte Retrieval

This study was approved by the Institutional Ethics Committee on Animal Use of the Universidade Estadual Paulista (Process n° 1803/17).

Ovaries (*n* = 174) from healthy queens (*Felis catus*) were harvested at random stages of the estrous cycle during routine ovariectomy at the unit of “Obstetrícia e Reprodução Animal” and in the “Centro de Esterilização de Caninos e Felinos” of the Universidade Estadual Paulista—Campus Jaboticabal, SP, Brazil.

After surgery, ovaries were immediately placed in a phosphate-buffered saline (PBS) with a mixture of antibiotics (AB) and antimycotics (100 IU/mL of penicillin G sodium, 0.1 mg/mL of streptomycin sulfate, 0.25 μg/mL of amphotericin B; Vitrocell Embriolife, Campinas, SP, Brazil), and transported to the laboratory at room temperature (RT) until processing (within 4 h).

Ovaries were minced in PBS and AB with 0.1% (*w*/*v*) polyvinyl alcohol to release oocytes, and only immature COCs (*n* = 414) with darkly pigmented ooplasm completely surrounded by one or more layers of cumulus cells (Grade I [27]) were selected for the experiments. For each experiment, COCs from different cats were pooled.

### 2.3. Experimental Design

In this study, fresh COCs were vitrified by the Cryotop method, and viable VOs after warming were in vitro matured in the 3D system (barium alginate microcapsules immersed in maturation medium) or the 2D system (traditional microdrops of medium, covered by mineral oil) for 24 h in a controlled atmosphere (38.5 °C and 5% CO_2_ in air), as follows:

− VOs in 3D coculture: VOs (*n* = 76) cultured in the 3D system in association with fresh COCs (i.e., COCs in 3D coculture, *n* = 77);
− VOs cultured separately in 3D (*n* = 75);
− VOs cultured separately in 2D (*n* = 77);
− COCs cultured separately in 2D (fresh control group, *n* = 87).

The cocultured groups (VOs in 3D coculture and COCs in 3D coculture) were obtained combining VOs and COCs in the same 3D microcapsule during in vitro maturation (IVM) in a 1:1 ratio.

At the end of IVM, in vitro fertilization (IVF) with fresh epididymal feline spermatozoa was performed, and presumptive zygotes were in vitro cultured in the 3D or 2D system, according to the IVM conditions. Embryonic development was recorded over seven days of culture.

### 2.4. Vitrification and Warming of Immature Cumulus-Oocyte Complexes (COCs)

Cat COCs collected from 112 ovaries were vitrified by the Cryotop method, as previously described by Kuwayama [28] and Cobo and colleagues [29]. Briefly, groups of 3–8 fresh immature COCs were equilibrated at RT in an equilibration solution (ES) containing 7.5% (*v*/*v*) ethylene glycol (EG) and 7.5% dimethylsulfoxide (Me2SO) in TCM199, with 20% fetal bovine serum (FBS). Then, they were transferred into a vitrification solution (VS: 15% (*v*/*v*) EG, 15% Me2SO and 0.5 M sucrose in TCM199 with 20% FBS), placed on Cryotop polypropylene strip, removing the excess of liquid to reduce the volume as much as possible, and directly immersed into liquid nitrogen. At warming, the Cryotop strip was immersed for 1 min in a thawing solution (TS) at 37 °C containing 1 M sucrose in TCM199, with 20% FBS. Vitrified oocytes were retrieved and transferred into solutions containing decreasing concentrations of sucrose (0.5 M and 0 M). Then, they were washed twice in TCM199 with 20% FBS, and transferred into fresh culture medium for the experiments.

### 2.5. Viability Assessment

After warming, to exclude oocytes that did not survive vitrification, VOs viability was evaluated by fluorescein diacetate/propidium iodide (FDA/PI) staining before starting IVM. Briefly, VOs were maintained in the dark in the staining solution (PI: 10 mg/mL; FDA: 5 mg/mL) for 3 min and then evaluated under a fluorescence microscope (Olympus IX70, Tokyo, Japan). Dead VOs (red fluorescence) were discarded, whereas viable VOs (green fluorescence) were washed with fresh culture medium and divided into the different experimental groups.

### 2.6. In Vitro Maturation in Three-Dimensional (3D) and Two-Dimensional (2D) Systems

Fresh and vitrified-warmed feline oocytes were in vitro matured in different culture conditions (2D, 3D, 3D coculture) in TCM199 supplemented with 3 mg/mL bovine serum albumin (BSA), 10 ng/mL epidermal growth factor (EGF), 0.6 mM cysteine, and 0.5 IU/mL FSH + 0.5 IU/mL LH (Pluset^®^, Calier, Spain). Fresh oocytes were collected on the same day as the IVM from 62 ovaries.

For the 3D system, a two-step encapsulation technique in barium alginate was developed, as described by Morselli and co-workers [22,23]. Briefly, the sodium-alginate powder (0.5%) was dissolved into sterile water and filtered to obtain the melting solution (MS) at medium viscosity (3500 cP, centipoise). A saturated solution of BaCl_2_ was then added to an aliquot of TCM199 to obtain the dropping solution of BaCl_2_ (40 mM), that was dropped at RT into the MS maintained stirred for 30–40 min. Resulting microcapsules were collected, washed twice in PBS and suspended in the maturation medium or maintained in PBS at 4 °C in a Petri dish until use.

Cat oocytes (VOs and COCs) were injected into the inner core of the microcapsules by a small bore glass pipette (6–14 gametes/microcapsule) and subsequently immersed in 1 mL of maturation medium in a multiwell plate.

For the 2D system, traditional microdrops of maturation medium (100 μL containing 4–10 oocytes) were placed in a Petri dish and covered by mineral oil.

### 2.7. Epididymal Sperm Recovery, In Vitro Fertilization, and Embryo Culture

In vitro fertilization was performed with fresh feline epididymal spermatozoa obtained after routine orchiectomy of adult tomcats (*n* = 32). The epididymides were dissected from isolated testicles and placed in a Petri dish in IVF medium, containing 6 mg/mL BSA, 2 mM L-glutamine, 0.36 mM pyruvate, 1.11 mM calcium lactate, 100 U/mL penicillin G Na, 0.05 mg/mL streptomycin, 8% (*v*/*v*) sterile water, and 0.05% phenol red in Tyrode’s solution (Vitrocell Embriolife, Campinas, SP, Brazil). Spermatozoa were obtained from vas deferens and cauda epididymis by direct squeezing in IVF medium under a stereomicroscope (Nikon SMZ745T, Tokyo, Japan), and subjective motility and concentration by Bürker chamber were determined.

**Figure 1:**
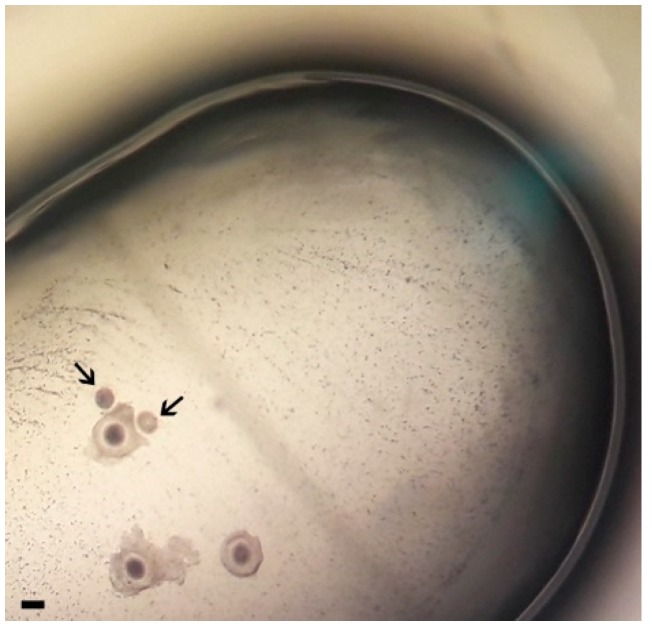
Domestic cat vitrified-warmed oocytes (VOs, black arrows) cocultured with fresh cumulus oocyte complexes (COCs) in a barium alginate microcapsule (three-dimensional (3D) system) during in vitro maturation (scale bar 100 µm).

After 24 h of IVM, oocytes were removed from microcapsules (by a small bore glass pipette) and microdrops, then washed twice and transferred into 50 μL drops of fresh IVF medium covered by mineral oil. VOs in 3D coculture and COCs in 3D coculture (Figure 1) were separated at this stage and placed in different IVF drops. Diluted sperm (2 × 10^6^ motile spermatozoa/mL) were added to the fertilization drops containing the oocytes.

At 18–24 h post insemination (Day 1), the oocytes were gently washed in in vitro culture (IVC) media (IVC1, reported below) to remove unbound spermatozoa and residual cumulus cells by a small bore glass pipette, and the presumptive zygotes were in vitro cultured for seven days in the 3D or 2D system, according to the IVM conditions, in IVC media (modified from [30], IVC1 (Day 1): 3 mg/mL BSA, 1% (*v*/*v*) non-essential aminoacids, 2 mM L-glutamine, 0.36 mM pyruvate, 1.11 mM calcium lactate, 0.5 μg/mL gentamicin, 0.004% amphotericin B, 8% sterile water and 0.02% phenol red in Tyrode’s solution; IVC1 + EAA (Day 2): 1% (*v*/*v*) essential aminoacids in IVC1; IVC2 (Days 3, 5, 7): 10% (*v*/*v*) FBS, 1% non-essential aminoacids, 2% essential aminoacids, 2 mM L-glutamine, 0.36 mM pyruvate, 1.11 mM calcium lactate, 0.5 μg/mL gentamicin, 0.02% amphotericin B, 8% sterile water and 0.02% phenol red in Tyrode’s solution).

For the creation of 3D alginate microcapsules for embryo culture, the same protocol for the 3D IVM system was applied, but Tyrode’s solution was used to obtain the dropping solution. Presumptive zygotes were injected into the inner core of the microcapsules by a small bore glass pipette and subsequently immersed in 1 mL of IVC medium in a multiwell plate. Two-dimensional culture was carried out in traditional microdrops (100 μL) of IVC media covered by mineral oil in Petri dishes. Embryonic development was recorded on days 1, 2, 3, 5, and 7 of culture.

### 2.8. Assessment of Maturation and Embryonic Developmental Rates

Two days after IVF, chromatin configuration of uncleaved oocytes were evaluated by a bis-benzimide (Hoechst 33342) staining. Growing embryos were cultured until they showed signs of degeneration or until day 7 and then stained with bis-benzimide to confirm their developmental stage based on the number of blastomere nuclei. In some cases, oocytes and embryos were stored in a blocking solution (1 mg/mL BSA, 100 mM glycine and 0.2% (*w*/*v*) sodium azide in PBS) at 4 °C until fluorescent analysis.

Oocytes and embryos, deprived of cumulus cells and bound spermatozoa (if any were remaining after washing from IVF to IVC1 medium) by mechanical displacement with a small bore glass pipette, were placed on a slide with a minimum amount of medium and then covered with the working solution (Hoechst, 0.01 mg/mL). After 5 min of incubation in the dark, the Hoechst solution was removed, the slides were covered with an anti-fade reagent (Fluoromount™ Acqueous Mounting Medium), and nuclear evaluation under a fluorescence microscope (Olympus IX70, Tokyo, Japan) at 400× magnification was performed, considering that the Hoechst 33342 maximum excitation wavelength is 352 nm and the emission wavelength is 461 nm.

Chromatin configurations of unfertilized oocytes were classified as follows [31,32]:

− germinal vesicle (GV): identification of nucleolus and very fine filaments of chromatin;
− germinal vesicle break-down–anaphase I (GVBD–AI): identification of different patterns of chromatin condensation (GVBD) or identification of bivalents (AI);
− telophase I–metaphase II (TI–MII): identification of two groups of chromosomes moving to opposite ends of meiotic spindle (TI) or two sets of chromosomes clearly visible (MII);
− degenerated: collapsed nucleus or irregular nuclear conformation.

Total maturation rates were calculated as the sum of unfertilized MII oocytes and cleaved embryos. For the assessment of embryonic development, cleaved embryos, 8–16 cells, morulae, and blastocysts stages were recorded.

### 2.9. Statistical Analysis

Data from seven replicates are reported as percentages (mean ± standard deviation (SD)). Maturation and embryonic developmental rates of oocytes were analyzed by one-way ANOVA followed by Tukey’s test for multiple comparisons. Before ANOVA, the proportions in every treatment group for each experiment were transformed by the cosine transformation, following the Formula (1), where y′ is the transformed value and y stands for the original proportion of success in a given variable:

y′ = arcsin√y(1)

The level of significance was set at *p* < 0.05.

## 3. Results

In this study, 250 cat COCs were vitrified, and immediately after warming only the viable oocytes (91.2%) were used for the experiments.

Proportions of maturation rates and embryonic developmental stages are reported in Table 1. Three-dimensional barium alginate microcapsules used during the in vitro embryo production (IVEP) were able to sustain the maturation and the embryonic development of feline VOs in all of the treatment groups, and their ability to resume meiosis and reach the late embryo stages (morulae and blastocysts) was observed.

Although the developmental potential of VOs was similar in all of the tested conditions, without remarkable differences between the culture in the 3D or 2D system, the only treatment in which the blastocyst stage was obtained by VOs was 3D microcapsules in association with companion COCs (one blastocyst, 0.95 ± 2.52%). Indeed, VOs cultured separately in 3D or 2D systems did not progress beyond the morula stage.

Fresh COCs developed at higher rates compared to VOs, both as companion cells (COCs in 3D coculture) or as the control group (COCs cultured separately in 2D), without differences among culture conditions (COCs in 3D coculture versus COCs cultured separately in 2D, *p* = 0.98).

Cleavage (2–4 cells embryos) rate was similar in 3D and 2D systems both among VOs (*p* = 1.00) and among COCs (*p* = 0.98). The same trend was observed for 8–16 cells embryos, that did not have significantly different values among neither VOs (*p* = 0.98) nor COCs (*p* = 0.57). Again, morulae/blastocyst rates were similar in 3D and 2D systems, and the development of VOs cultured separately in 2D was similar to that of COCs in 3D coculture (*p* = 0.37), whereas VOs in 3D coculture and VOs cultured separately in 3D developed at lower rates compared to fresh COCs (*p* = 0.04).

Nuclear configurations of oocytes that did not develop into embryos were also assessed but no differences were observed, neither between VOs and fresh COCs nor between 3D and 2D culture systems. The proportion of immature oocytes (GV) was similar in 3D and 2D systems among VOs and COCs (*p* = 0.33), as well as the proportion of oocytes that partially resumed meiosis (GVBD–AI; *p* = 0.74).

## 4. Discussion

In this study, the suitability of a 3D enriched system (barium alginate microcapsules alone or in association with fresh companion COCs during IVM) used for the IVEP of vitrified immature domestic cat oocytes was investigated by assessing their embryo development. Vitrified oocytes achieve the full developmental competence at very low rates compared to fresh ones due to several cryoinjuries, which dramatically impair their maturation ability after warming [2,3]. Although cryopreservation has been proven more efficient for mature than immature female gametes [33], developing protocols for immature cryopreserved oocytes would be very promising for assisted reproductive techniques because this source of genetic material might be more readily (or the only one) available, especially in field conditions. Therefore, the application of enriched culture systems to recreate a more adequate microenvironment for stressed and low-competence gametes could be advisable.

Present data show that domestic cat VOs retained their intrinsic developmental potential after warming, as full nuclear maturation and fertilization were obtained. In all treatment groups, VOs were able to mature and develop until late embryo stages (morulae or blastocysts) although, as expected, at lower rates than fresh high-competence COCs, used as companion cells or as the control group. The proportions of cleavage and morulae and blastocyst stages were similar to previous studies, in which Open Pulled Straws or Cryotop vitrification protocols were used [34,35,36].

The challenge of increasing the proportions of VOs late embryo stages relies on overcoming the cold-induced damages that unavoidably involve both the inner and the outer compartments of the oocytes. In this study, although more than 90% of VOs were viable at warming, a dramatic decrease of their developmental potential occurred over in vitro culture. Indeed, in other species, a remarkable decline of oocytes viability was observed 2 h after the restoration of physiological conditions [37], underlining that most of the cryodamage effects appear some time after warming and cannot be properly identified before starting culture.

Since the extracellular environment might positively influence the intracellular conditions [21], the culture of VOs in a 3D microenvironment, more similar to the follicular niche, was tested. Although the in vitro development of immature VOs after warming is still a challenge, in this study VOs were able to mature and be fertilized, and similar maturation and embryonic developmental rates were obtained in both 3D and 2D conditions (*p* > 0.05), pointing out that 3D barium alginate microcapsules, closer to physiological conditions, are suitable for the culture of VOs, although not enough for these highly demanding gametes. Alginate is a natural biomaterial, with high biocompatibility and low toxicity [38], appropriate for both oocytes IVM and embryonic development in different species (mouse [13], cow [17], cat [22,23]). The microcapsule conformation ensures a physiological spatial organization of the oocytes and the maintenance of metabolic activity [39,40]: alginate permeability allows for the exchange of nutrients, growth factors, and hormones through the microcapsules to the enclosed oocytes/embryos, such as in the follicular/oviductal environments.

However, for their structure, 3D microcapsules and 2D microdrops had to employ different volumes of culture medium, and this difference might have influenced the results of embryo development. In the domestic cat, a very small volume (20 μL) negatively affected the progression to late embryo stages compared to bigger droplets (50 and 100 μL, like those used in the present study) [41], whereas in other species smaller volumes were more favorable for embryo development (mouse [42,43]) or exerted no effect (human [44]), but the volumes tested vary and the matter would need further investigation.

This is the first experiment of a 3D microenvironment for the in vitro culture of cryopreserved oocytes and derived embryos, as previous reports were only focused on cryopreserved follicles or tissue [45,46,47,48,49,50,51,52,53,54,55,56,57,58]. The present results showed that the 3D environment alone did not properly promote the in vitro competence of VOs when cultured separately, but the association with cocultured COCs was the only condition able to allow VOs to achieve the blastocyst stage. The supplementation of external stimuli provided by the active high-competence COCs during IVM might supply chemical factors involved in the acquisition of competence that VOs themselves are no longer able to self-produce. No previous studies have investigated similar enriched conditions (3D coculture) for feline VOs during IVM, and only Attanasio and colleagues [26] performed the coculture of buffalo VOs with fresh competent COCs during IVF, reporting an increase in cleavage rates. Similarly, the preservation of VOs somatic compartment, crucial for the acquisition of developmental competence [58], or its replacement with fresh granulosa/cumulus cells might be helpful to supply important factors to fully exploit VOs potential, since cryoinjuries severely impair the functional connections between the somatic and the germinal compartments, causing a failure in meiosis resumption and consequently in the progression to late embryo stages [59,60].

Moreover, although the presence of companion oocytes is known to be beneficial for the positive effects of exchanged molecules during coculture, as expected in this study associated COCs in 3D microcapsules developed at similar rates to those alone in 2D microdrops, underlining that the coculture in the same environment with VOs was not as beneficial as reported by other authors who employed other low-competence (i.e., fresh denuded) oocytes as companion cells [19,22,61]. One hypothesis might be that, after warming, the intracellular molecular machinery of VOs was unable to properly carry out basal activities, such as the secretion of paracrine factors (e.g., oocyte-secreted factors) involved in the acquisition of the developmental competence. Investigating the expression profile of VOs or detecting their secreted proteins in the culture medium might be helpful to assess their transcriptional activity and protein synthesis ability, and thus to target more specifically their deficiencies in a custom-made culture system.

As a strategy for the improvement of VOs outcomes through the enhancement of intercellular chemical signaling, the coculture with proliferating somatic cells (i.e., granulosa cells) could be tested, since homologous or heterologous granulosa cells can supply steroid hormones and signaling molecules, regulating meiotic progression and supporting cytoplasmic maturation [62]. Granulosa cells cultured in 3D barium alginate microcapsules could recreate a follicle-like structure in vitro [63] and resemble more closely the architecture and the signaling pathways of follicles, giving low-competence gametes the best chances to develop into embryos properly.

## 5. Conclusions

In conclusion, the enriched culture conditions (3D alginate microcapsules alone or in association with fresh competent COCs during IVM) proposed for the in vitro embryo production of immature cat VOs for the first time in this study were able to support their in vitro maturation and embryo development, although these gametes experienced cryodamages that often hindered their developmental competence. However, the enriched culture conditions were comparable to 2D environments, for what concerns the developmental rates of VOs and improvements are still needed. Further investigations should be carried out to increase the proportions of late embryo stages, and bringing together living somatic cells and 3D matrices for the creation of an artificial and viable follicle-like structure could be promising.

## Figures and Tables

**Table 1:** Maturation and embryonic developmental rates of vitrified and fresh feline oocytes following in vitro embryo production in the three-dimensional (3D) or two-dimensional (2D) system. Data reported as percentages (mean ± standard deviation (SD)). Different superscripts (^a,b,c^) indicate significant differences among groups (*p* < 0.05). Proportions calculated on the total number of oocytes in each group. VOs: vitrified oocytes; COCs: fresh oocytes; 3D system: barium alginate microcapsules; 2D system: microdrops of medium; M: morulae; BL: blastocysts; GV: germinal vesicle; GVBD: germinal vesicle break-down; AI: anaphase I. VOs in 3D coculture: VOs matured in 3D system in association with fresh COCs (i.e., COCs in 3D coculture) and cultured in 3D system after in vitro fertilization; VOs cultured separately in 3D: VOs matured in 3D system without fresh companion COCs and cultured in 3D system after in vitro fertilization; VOs cultured separately in 2D: VOs matured in 2D system without fresh companion COCs and cultured in 2D system after in vitro fertilization; COCs in 3D coculture: COCs matured in 3D system in association with VOs (i.e., VOs in 3D coculture) and cultured in 3D system after in vitro fertilization; COCs cultured separately in 2D: COCs matured in 2D system and cultured in 2D system after in vitro fertilization as fresh control group.

Groups	Maturation ^1^	Embryonic Development				Degeneration	Unfertilized Oocytes	
		Cleavage (2–4 cells)	8–16 cells	M + BL	(M + BL)/Cleaved		Immaturity (GV)	Meiosis Resumption (GVBD–AI)
VOs in 3D coculture (*n* = 76)	21.25 ± 18.98 ^a^	18.39 ± 16.67 ^a^	7.39 ± 7.94 ^a^	1.79 ± 3.07 ^a^	9.52 ± 16.27 ^a,b^	58.23 ± 28.51	4.65 ± 7.39	15.87 ± 14.43
VOs cultured separately in 3D (*n* = 75)	19.46 ± 13.71 ^a^	17.42 ± 11.83 ^a^	4.59 ± 9.38 ^a^	1.79 ± 4.72 ^a^	4.76 ± 12.60 ^b^	59.00 ± 21.21	8.38 ± 12.92	13.16 ± 14.91
VOs cultured separately in 2D (*n* = 77)	21.89 ± 11.98 ^a^	14.96 ± 8.80 ^a^	8.04 ± 5.82 ^a^	5.97 ± 7.27 ^a,b^	38.89 ± 49.07 ^a,b^	60.15 ± 19.19	10.48 ± 10.60	7.48 ± 9.34
COCs in 3D coculture (*n* = 77)	47.44 ± 17.47 ^b^	44.58 ± 17.98 ^b^	33.00 ± 12.18 ^b^	16.76 ± 14.97 ^b,c^	39.22 ± 29.55 ^a,b^	37.27 ± 24.88	0.84 ± 2.22	14.44 ± 15.11
COCs cultured separately in 2D (*n* = 87)	52.16 ± 14.38 ^b^	50.88 ± 13.71 ^b^	46.23 ± 15.15 ^b^	25.68 ± 7.91 ^c^	52.49 ± 17.56 ^a^	36.46 ± 11.79	4.12 ± 6.44	7.26 ± 9.20

^1^ Sum of cleaved embryos and unfertilized metaphase II oocytes.
